# Systematical Study on the Influencing Factors of Synchronous Thermal Analyses of Samples-Taking the Chalcanthite as an Example

**DOI:** 10.3389/fchem.2022.863083

**Published:** 2022-04-13

**Authors:** Duan Xianzhe, Li Nan, Wang Yuyuan, Tang Zhenping

**Affiliations:** ^1^ School of Resource & Environment and Safety Engineering, University of South China, Hengyang, China; ^2^ Hunan Key Laboratory of Rare Metal Minerals Exploitation and Geological Disposal of Wastes, Hengyang, China; ^3^ College of Chemistry and Chemical Engineering, University of South China, Hengyang, China

**Keywords:** synchronous thermal analyzer, thermogravimetric analyses, differential thermal analyses, chalcanthite, influencing factors

## Abstract

Thermal analysis is widely used for the measurement of the relationship between temperature and physical properties of the materials. Many studies have reported different thermal analysis methods, including thermogravimetry (TG), derivative thermogravimetry (DTG), differential heat analysis (DTA), and differential scanning calorimetry (DSC), but few comprehensively studied the factors influencing TG-DTA by the combined thermogravimetry–differential thermal methods. In this study, taking chalcanthite as the research object, the thermogravimetric–differential thermal analyses were systematically conducted by using synchronous thermal analyzer technology. The results demonstrate that 1) DTA curves of low- and medium-weight chalcanthite show five dehydration endothermic peaks, while TG curves do not display obvious weight-loss steps; DTA and TG curves of high-weight chalcanthite samples, on the other hand, illustrate three endothermic peaks, indicating three-step loss of crystalline water; 2) higher weight of samples may cause longer time of internal heat transfer and larger temperature gradient, consequently resulting in the expansion of DTA peak shape and the decline of resolution as well as the increase of the peak temperature; 3) the weight-loss deviation between the measured and theoretical data is relatively higher in the low-weight samples than that in the medium- and high-weight samples; 4) the heating rate can increase the DTA curve peak and thermal inertia and the temperature at the thermodynamic equilibrium, causing the temperature lagging behind and the overall peak moving toward high temperature; 5) sample grinding may destroy the structure of the crystal, thereby breaking the relatively weak chemical bond, and thus affects the structure of thermogravimetric–differential thermal analyses. These suggest that the sample weight, heating rate, and sample grinding probably have significant effects on the thermogravimetric–differential thermal analyses. Therefore, proper experimental conditions are needed to obtain the accurate results during the thermogravimetric–differential thermal analyses. This study can provide a basis and reference for future synchronous thermal analyses.

## Introduction

Thermal analysis is a technique for measuring the relationship between temperature and physical properties of the materials at various temperatures controlled by different processes (Chihara, 1999). Many studies have reported different thermal analysis methods, including thermogravimetry (TG), derivative thermogravimetry (DTG), differential heat analysis (DTA), and differential scanning calorimetry (DSC), which are widely used to study the physical and chemical changes of materials and obtain important information about materials including composition, stability, chemical reaction process, and physicochemical and thermodynamic properties (Liu and Shan, 2000, 2006; Zhao et al., 2001; Bhosekar et al., 2006; Chaudhary et al., 2013a; Chaudhary et al., 2015; Chaudhary et al., 2016; Charmas et al., 2019; EL-Sayed and Mostafa, 2021; Meireles et al., 2021). There are many factors affecting the thermogravimetry curve, such as the crucible and instrument sensitivity as well as the effects of operating conditions (e.g., sample weight, heating rate, granularity, and filling conditions) (Gao et al., 2002; Prabhumirashi and Khoje, 2002; Liu et al., 2006; Vlaev et al., 2008). However, few studies comprehensively studied the factors influencing TG-DTA by the combined thermogravimetry–differential thermal methods. Compared with separate TG or DSC methods, the synchronous thermal analyzer has the following significant advantages: 1) it can eliminate the negative influences of weighing, sample uniformity, heating rate consistency, atmospheric pressure, and flow difference; 2) the TG and DTA curves have much better correspondence, making the results more accurate.

Chalcanthite, a typical crystalline aquo-compound, is widely used in electroplating, textile printing, pigments, and pesticides (Chen, 2014a). It is a good research object to investigate the influencing factors of synchronous thermal analyses. Many studies have reported differential thermal (Pan et al., 1988), thermogravimetry (Chen and Yu, 2001; Li et al., 2008; Chen, 2014b), and synchronous thermal (Lu et al., 2001) analyses of chalcanthite, but the factors affecting the thermogravimetric–differential thermal analyses remain unclear. In this study, we systematically investigate the effects of sample weight, heating rate, and sample grinding on the thermogravimetry–differential heat analyses of chalcanthite with an aim to provide a basis and reference for future synchronous thermal analyses.

## Materials and Methods

### Experimental Reagent

Analyzed pure chalcanthite and alumina crucible were used.

### Experimental Instrument

The HCT-4-type synchronous thermal analyzer (Beijing Hengjiu Experimental Equipment Co., Ltd.) was used.

### Experimental Methods

#### Contrast Experiments With Different Weight of Samples

Chalcanthite crystals of 5.2, 9.6, and 28.7 mg were accurately weighed and then put in the alumina crucible, with the empty alumina crucible as the reference. The temperature was set from 25 to 400°C in the air atmosphere at the heating rate of 5°C/min, maintaining 400°C for 5 min, and the samples were then collected to obtain the thermogravimetric–differential thermal analysis spectrogram.

#### Contrast Experiments at Different Heating Rates

Accurately weighed three chalcanthite samples of 9.6 mg were put in the alumina crucible, with the empty alumina crucible as the reference. The temperature was raised from 25 to 400°C in the air atmosphere at the heating rates of 3, 5, and 10°C/min. The final temperature of 400°C was maintained for 5 min. The thermogravimetric–differential thermal data were finally obtained for the samples collected.

#### Contrast Experiments With Ground and Non-Ground Chalcanthite Samples

Two ground and non-ground chalcanthite samples of 9.6 mg were weighed and then placed in the alumina crucible, with the empty aluminum crucible as the reference. The temperature was raised from 25 to 400°C at the heating rate of 5°C/min in the air atmosphere, keeping the time for 5 min, and the thermogravimetric–differential thermal analyses were conducted for the samples obtained.

## Results and Discussion

### Effect of the Sample Weight on the DTA, TG, and DTG Spectrograms

**FIGURE 1:**
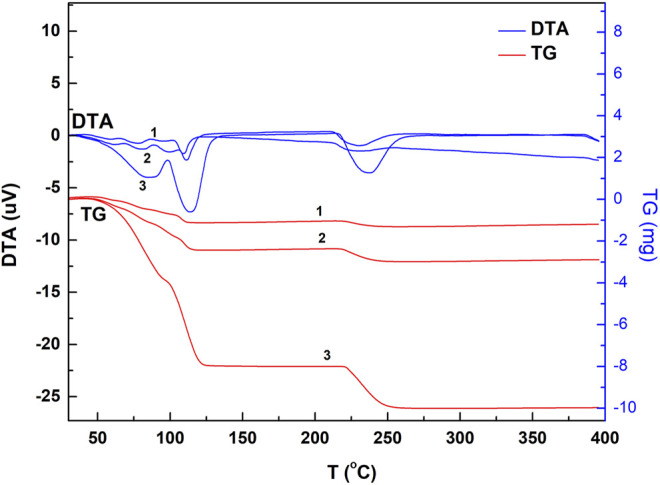
DTA and TG spectrogram of the chalcanthite crystals of different weight. Note: upper curves 1, 2, and 3 are DTA profiles of low-, medium-, and high-weight chalcanthite crystals, respectively, while lower curves 1, 2, and 3 are TG profiles of low-, medium-, and high-weight chalcanthite crystals, respectively.

**FIGURE 2:**
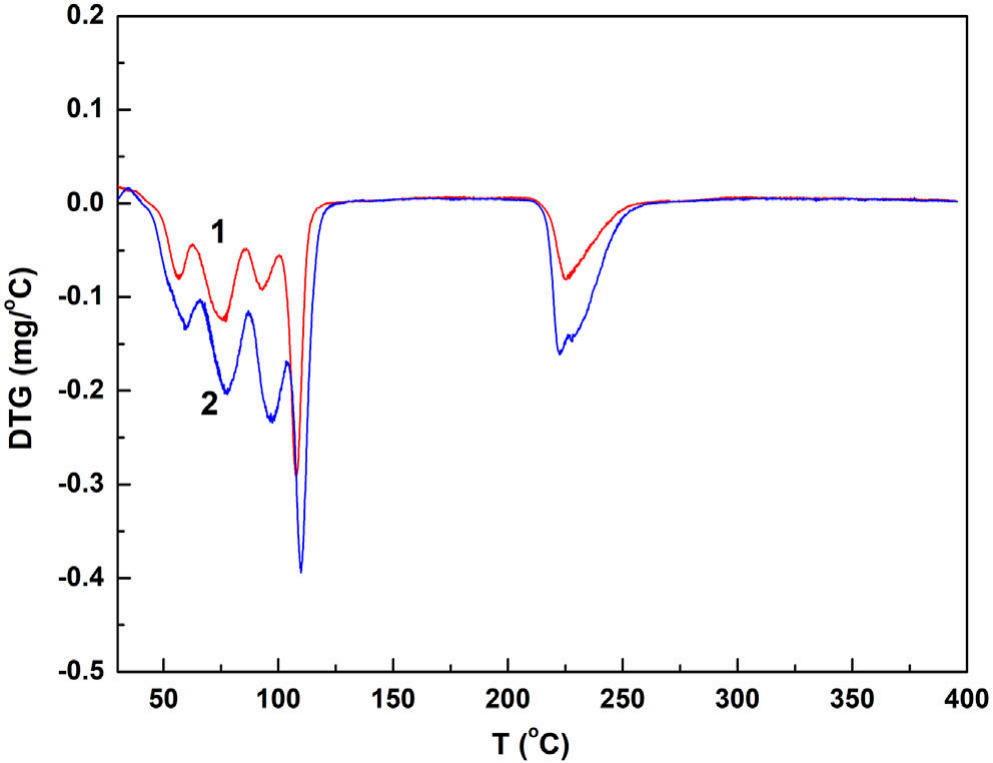
DTG spectrogram of the low- and medium-weight chalcanthite crystals. Note: curves 1 and 2 are DTG profiles of low- and medium-weight chalcanthite crystals, respectively.

Chalcanthite crystals of 5.2, 9.6, and 28.7 mg were collected, respectively, with the corresponding results of DTA, TG, and DTG obtained (Figures 1, 2;Table 1).

**TABLE 1:** Peak temperature data of chalcanthite with different weights obtained according to the thermogravimetry curve corresponding to the differential thermal peak.

	Peak temperature in the first step (T_m_/°C)	Peak temperature in the second step (T_m_/°C)	Peak temperature in the third step (T_m_/°C)	Peak temperature in the fourth step (T_m_/°C)	Peak temperature in the fifth step (T_m_/°C)
Low weight	58.60	77.88	96.19	109.04	230.87
Medium weight	61.57	80.30	100.03	111.06	230.33
High weight	85.63	113.87	237.09	—	—

Note: T_m_ represents the peak temperature.

From Figure 1; Table 1, it can be seen that the DTA curves of low- and medium-weight chalcanthite show five dehydration endothermic peaks, while the TG curves do not show the obvious weight-loss step. In addition, the peak temperature of DTA curves increases with the weight of samples during each weight-loss step (Table 1). However, the DTG curves of low- and medium-weight chalcanthite crystals show five maximum reaction rate peaks, indicating that the crystalline water of these crystals is lost in five steps (Figure 2). A total of three endothermic peaks in DTA curves and three obvious steps in TG curves of high-weight chalcanthite samples indicate that the crystalline water is lost in three steps in the high-weight case (Figure 1; Table 2). These altogether suggest that increasing weight of samples probably increases the internal heat transfer time and the temperature gradient, consequently resulting in the expansion of DTA peak shape and the decline of resolution as well as the increase of the peak temperature (i.e., more significant temperature lag) (Chaudhary et al., 2012; 2013b). Therefore, the weight of the sample should reasonably be reduced within the sensitivity range of thermobalance (Song et al., 2011). For some materials with low thermal sensitivity and weight-loss rate, higher weight can be used, but the sample weight should be controlled within the sensitivity range of the thermogravimetric analyzer (Wang and Xiao, 2005).

**TABLE 2:** Weight-loss data of chalcanthite with different weights obtained according to the thermogravimetry curve corresponding to the differential thermal peak.

	Water loss in the first step %	Water loss in the second step %	Water loss in the third step %	Water loss in the fourth step %	Water loss in the fifth step %	Total weight loss %	Theoretical weight loss %
Low weight	3.68	8.08	4.45	7.54	5.07	28.82	36.08
Medium weight	5.70	7.04	7.01	6.05	6.39	32.19	36.08
High weight	13.81	13.85	6.93	—	—	34.59	36.08

The weight-loss data (Table 2) of the chalcanthite samples with different weight are obtained according to the thermogravimetry curve corresponding to the differential thermal peak. From Table 2, it can be seen that low-weight samples have relatively larger weight-loss deviation between the measured and theoretical data than the medium- and high-weight samples due to their higher sensitivity requirements of the comprehensive thermal analyzer.

### Effect of the Heating Rate on the TG-DTA Spectrogram

**FIGURE 3:**
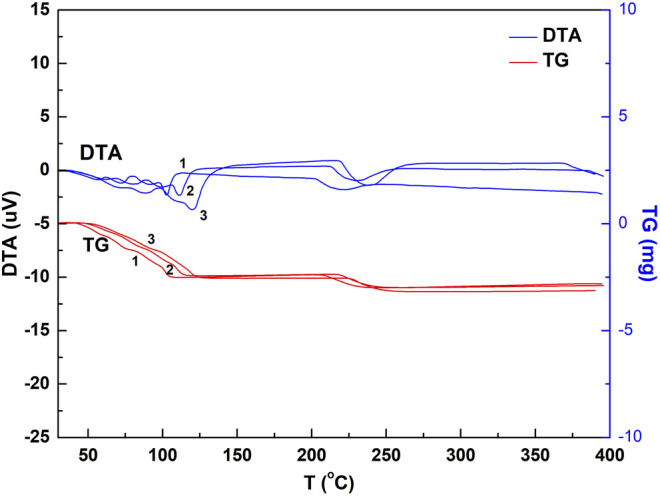
DTA and TG spectrogram of the chalcanthite crystals at different heating rates. Note: upper curves 1, 2, and 3 are DTA profiles of the chalcanthite crystals at heating rates of 3, 5, and 10°C/min, respectively, while lower curves 1, 2, and 3 are TG profiles of chalcanthite crystals at heating rates of 3, 5, and 10°C/min, respectively.

Chalcanthite samples of 9.6 mg were heated at 3, 5, and 10°C/min, respectively, with the corresponding results of DTA and TG obtained (Figure 3).

As seen in Figure 3, the heating rate increases the DTA curve peak, enhancing the peak sensitivity. This may be due to the temperature difference increased by the thermal effect per unit time (Chen, 2003). Table 3 shows that the heating rate increases the initial temperature extrapolated by water loss of each step. This may be attributed to the increase of thermal inertia by the heating rate. In addition, the shape of the curve also significantly changes by increasing the heating rate, that is, the peak becomes wider (Figure 3). The increasing heating rate results in the increase of the temperature at the thermodynamic equilibrium, accompanied by the temperature lagging behind and the overall peak moving toward high temperature. Nevertheless, the weight-loss value is almost not affected. While the appropriate heating rate is a key factor for improving the experimental accuracy, it is related to the sample’s nature. For samples with poor thermal conductivity, the heating rate can be appropriately reduced in the case of high instrument sensitivity; otherwise, it should be increased (Shen and Yang, 2005).

**TABLE 3:** Extrapolated initial temperature by water loss of chalcanthite at different heating rates.

Heating rate	First step water loss (T_e_/°C)	Second step water loss	Third step water loss	Fourth step water loss	Fifth step water loss
3°C/min	42.42	64.07	81.91	99.01	204.59
5°C/min	46.09	71.14	90.91	107.32	213.64
10°C/min	55.63	80.20	101.26	116.31	226.91

Note: T_e_ represents the initial temperature.

### Effect of the Sample Granularity

**FIGURE 4:**
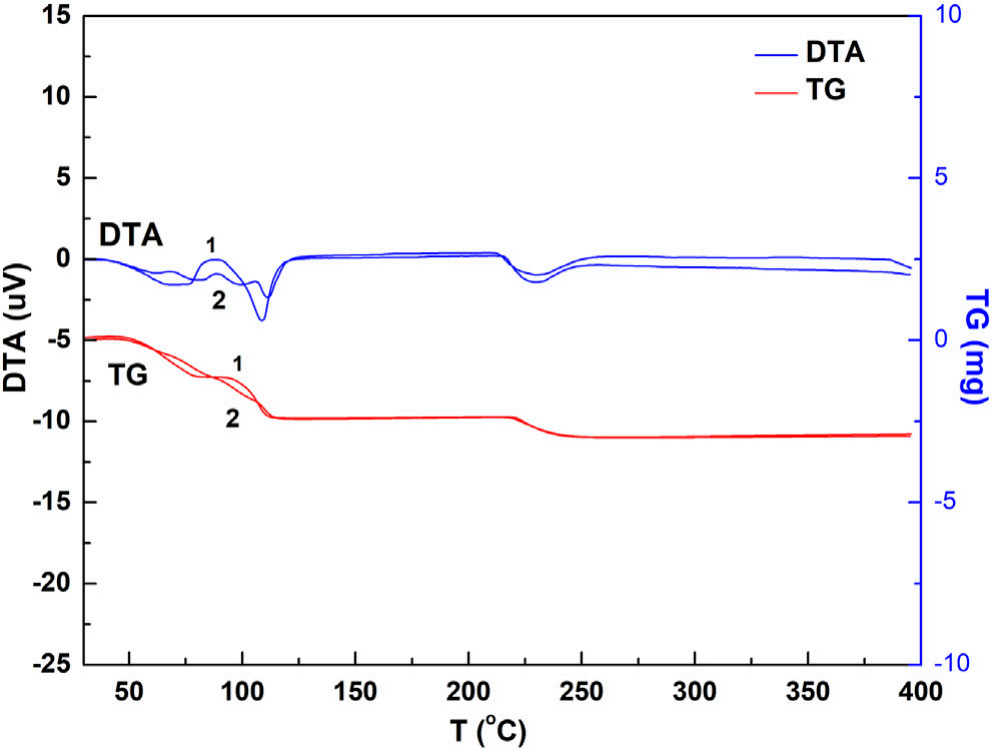
DTA and TG spectrogram of the chalcanthite crystals with and without grinding. Note: upper curves 1 and 2 are DTA profiles of ground and non-ground chalcanthite, respectively, while lower curves 1 and 2 are TG profiles of the ground and non-ground chalcanthite crystals, respectively.

The ground and non-ground chalcanthite samples of 9.6 mg were weighed, respectively, with the corresponding results of DTA and TG obtained (Figure 4).

Figure 4 shows three water-loss heat absorption peaks in the DTA curve and three obvious steps in the TG profile of the ground chalcanthite, indicating that the water is lost in three steps. This may demonstrate that the grinding may destroy the chalcanthite crystal structure, resulting in the break of the relatively weak bond. In addition, the grinding has a notable effect on the weight-loss stage, producing three obvious steps of the TG curve but an insignificant effect on the weight-loss value.

## Conclusion

In this study, taking chalcanthite as a research object, the synchronous thermal analyses under different conditions were conducted, and the following conclusions were drawn: 1) five dehydration endothermic peaks were obtained in the DTA curves of low- and medium-weight chalcanthite, whereas insignificant weight-loss steps were displayed in the TG curves; on the other hand, DTA and TG curves of high-weight chalcanthite samples exhibit three endothermic peaks, demonstrating three-step loss of crystalline water; 2) increasing weight of samples may cause longer time of internal heat transfer and larger temperature gradient, thus leading to the expansion of DTA peak shape and the decline of resolution as well as the increase of the peak temperature; 3) low-weight samples possess relatively larger weight-loss deviation between the measured and theoretical data than the medium- and high-weight samples; 4) the heating rate can increase the DTA curve peak, thermal inertia, and the temperature at the thermodynamic equilibrium, causing the temperature lagging behind and the overall peak moving toward high temperature; 5) sample grinding may destroy the structure of the crystal, thereby breaking the relatively weak chemical bond and consequently influencing the structure of thermogravimetric–differential thermal analyses; 6) proper experimental conditions should be considered for accurate measurements during the thermogravimetric–differential thermal analyses.

## Data Availability

The original contributions presented in the study are included in the article/Supplementary Material, further inquiries can be directed to the corresponding authors.
